# Lipidomic profiling of endometrial cancer using desorption electrospray ionization mass spectrometry imaging

**DOI:** 10.1073/pnas.2522839122

**Published:** 2025-11-24

**Authors:** Maria Paraskevaidi, Olivia Raglan, James McKenzie, Yuchen Xiang, Stefania Maneta-Stavrakaki, Maria Luisa Doria, Apostolia Galani, Nada Assi, Eftychios Manoli, Laura Burney Ellis, Baljeet Kaur, Francesca Rosini, Marc J. Gunter, Zoltan Takats, Maria Kyrgiou

**Affiliations:** ^a^Department of Metabolism, Digestion and Reproduction, Faculty of Medicine, Imperial College London, London W12 0NN, United Kingdom; ^b^West London Gynaecological Cancer Centre, Imperial College Healthcare National Health Service Trust, London W12 0HS, United Kingdom; ^c^Biostatistics Department, HEVA, Lyon 69006, France; ^d^Imperial College Healthcare National Health Service Trust, North West London Pathology, London W12 0HS, United Kingdom; ^e^School of Public Health, Faculty of Medicine, Imperial College London, London W12 0BZ, United Kingdom

**Keywords:** desorption electrospray ionization mass spectrometry imaging (DESI-MSI), endometrial cancer, lipidomics, proteomics

## Abstract

Novel technologies are required to improve endometrial cancer diagnosis and enhance the early detection of preinvasive precursors. Herein, desorption electrospray ionization mass spectrometry imaging (DESI-MSI) was used to differentiate malignant from benign endometrial tissues and assess lipidomic differences among patients with obesity and diabetes. Reverse phase protein array (RPPA) analysis was performed in the same patients to gain insights into the altered signaling pathways and investigate the protein expression levels in endometrial cancer cases and controls. Tissues from 64 women (50 cancer, 14 benign) were analyzed with DESI-MSI achieving 90% sensitivity and 93% specificity. Discriminatory spectral features were primarily phospholipids [phosphatidic acid (PA), phosphatidylethanolamine (PE), phosphatidylserine (PS), and phosphatidylinositol (PI)], all elevated in cancer. Proteomics revealed upregulated proteins linked to commonly dysregulated pathways in endometrial cancer, namely PI3K/AKT/mTOR, MAPK/RAS, and Wnt signaling pathways. Lipidomic differences were found between high- (obesity/diabetes) and low-risk phenotypes (no obesity/diabetes), with PE and PS elevated in high-risk benign and PE reduced in high-risk cancer cases. A single phospholipid (PE(O-38:4)) was found as discriminatory in both normal and cancer cohorts, which may serve as biomarker in women with benign histology at high-risk of developing endometrial cancer. This study reports the application of DESI-MSI for lipidomic characterisation of endometrial cancer.

## Significance

Endometrial cancer is rising globally, yet early detection remains a clinical challenge. We have applied desorption electrospray ionization mass spectrometry imaging (DESI-MSI) for lipidomic profiling of endometrial tissues, achieving 90% sensitivity and 93% specificity in distinguishing malignant from benign cases. Our findings reveal lipidomic alterations linked to obesity and diabetes and identify phospholipid PE(O-38:4) as a potential biomarker of cancer risk in histologically benign tissues. Combined with proteomic analysis, this work highlights key dysregulated pathways (PI3K/AKT/mTOR, MAPK/RAS, and Wnt) and lays the foundation for more objective, automated diagnostic tools. DESI-MSI could enhance risk stratification, diagnosis, and intraoperative decision-making, with immediate translational potential and implications for transforming clinical care.

Endometrial cancer is the most common gynecological cancer in the United Kingdom (1), with an increase of over 58% in incidence rates since the early 1990s (2). The rising incidence of endometrial cancer has been mainly attributed to increasing obesity (3) and diabetes (4), as the worldwide prevalence of obesity has more than doubled (5) and that of diabetes has more than quadrupled since 1990 (6). Lifestyle factors that have been suggested to increase the risk for endometrial cancer include a high-fat diet, physical inactivity, hormonal therapy, or the use of tamoxifen for breast cancer prevention and treatment (7). Modification and management of high-risk factors could lower the risk for developing endometrial cancer (8). Although there have been advances, prevention, diagnosis, and treatment of endometrial malignancy continue to present major challenges, with around a fifth of cases being diagnosed at a late stage and mortality rates expected to rise by 12% by 2040 (9).

Most cases of endometrial cancer are diagnosed early as women present with symptoms of abnormal uterine bleeding (10). Symptoms are commonly investigated with transvaginal sonography (TVS) with or without endometrial sampling with pipelle biopsy, however, there are still challenges in diagnosis. Although the accuracy of sonography has improved with advances in technologies, TVS continues to have a significant number of false positive and negative results depending on the endometrial thickness cut-off (11). Women diagnosed with endometrial malignancy, commonly undergo a hysterectomy and bilateral salpingo-oophorectomy with or without sentinel lymph node sampling or pelvic lymphadenectomy. The histological type, now including molecular classification, and the depth of myometrial invasion on histopathological examination are some of the factors that determine the stage of early disease and tailor adjuvant treatments with external beam radiotherapy, brachytherapy, and chemotherapy. However, histopathological assessment is observer-dependent, not automated, and requires staining and pathology exploration (12, 13).

To date, no interventions have been shown to be of value in the prevention of uterine neoplasms. Several histological features are frequently described in women with benign endometrial biopsies. Endometrial hyperplasia with or without atypia have been associated with various levels of risk for malignant transformation (14). Other features such as increased or disordered proliferation are frequently reported although their role in determining women at high risk of developing endometrial malignancy is less known.

Mass spectrometry technologies hold promise as automated and objective tools complemented with molecular information. These tools can describe different biological signatures within tissue and may offer advantages in diagnostic, therapeutic, and prevention challenges in endometrial malignancy (15). Mass spectrometry imaging (MSI) provides simultaneous spatial and molecular information for a biological sample, therefore providing the distribution and identity of biomolecules, such as proteins, lipids, and fatty acids (16). Widely used MSI approaches include secondary ion mass spectrometry (SIMS) and matrix-assisted laser desorption ionization (MALDI), both of which achieve high spatial resolution but require time-consuming sample preparation steps (17). Desorption electrospray ionization (DESI)-MSI is a technique that allows chemical information to be obtained directly from a wide range of surfaces and is becoming increasingly used in cancer studies as it allows faster, ambient surface sampling without the need for ionization matrices or laborious sample preparation steps (18, 19). DESI is a soft ionization technique where primary electrically charged aqueous solvent droplets and ions of solvent are directed onto the surface of a sample, causing desorption of the sample’s surface, and thus generating secondary gas-phase ions originally present on the sample (19). The desorbed gaseous ions are subsequently transferred to a conventional mass spectrometer via an atmospheric pressure ion-transfer line for analysis (19). Carcinogenesis is well associated with altered lipid metabolism and this dysregulation can be detected by DESI-MSI (20). DESI-MSI is a nondestructive, label-free imaging technique which has been employed for the investigation of numerous diseases, such as endometriosis (21), and different types of cancer, such as liver (22), prostate (23), colorectal (24), gastric (25), brain (26), ovarian (27), breast (28–30), esophageal (31), and lung (20). More specifically, previous DESI-MSI studies have reported classification of endometriosis with an overall accuracy of 99%, whereas the lung, breast, gastric, esophageal, and ovarian cancers have been detected with diagnostic accuracies higher than 85% (20, 25, 27, 30, 31).

**Fig. 1.**
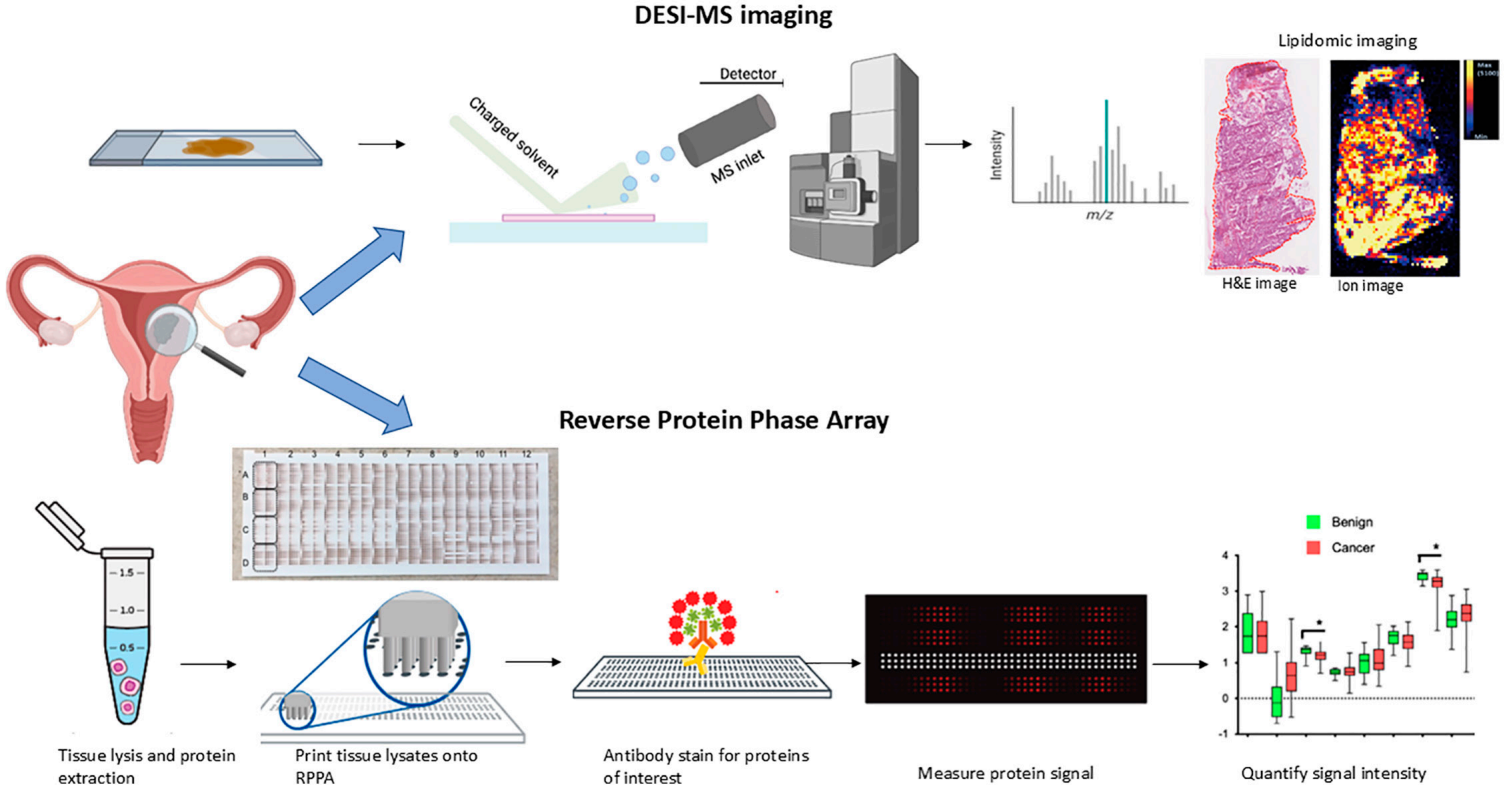
Schematic of the workflow used to perform DESI-MSI and RPPA analysis. For DESI-MSI, endometrial samples were cryosectioned and deposited on glass slides for analysis. The same tissue section was stained with hematoxylin and eosin (H&E) for histopathological diagnosis. Coregistration of the histological image to the DESI-MS image enabled annotation of the regions of interest. For RPPA, tissue samples were homogenized in lysis buffer and tissue lysates were serially diluted and arrayed on nitrocellulose-coated slides. Samples were probed with validated primary antibodies, specific for the protein of interest, and slides were scanned to analyze the spot intensity and determine protein levels or phosphorylated protein counterparts for each sample.

In this study, we explored whether DESI-MSI could be used as an objective, user-independent and automated tool to permit assessment of endometrial tissue and simultaneously provide valuable molecular information. We assessed the diagnostic accuracy of the technology in distinguishing endometrial cancer from benign control samples and described the discriminatory spectral features between the groups. We further explored the impact of obesity and diabetes on the phospholipid signature in benign and malignant endometrial tissues to assess whether individuals with changes in tissue carried a “high-risk” lipidomic profile similar to that of the malignant cases. Last, we performed protein expression studies using reverse phase protein array (RPPA) analysis to delve more into the dysregulated biological pathways in endometrial cancer and support our DESI-MSI lipidomic data (Fig. 1). RPPA analysis enabled the investigation of protein expression levels in endometrial cancer and benign tissues, which can be linked to the biology of cancer progression. This includes identifying the role of significantly expressed proteins in fundamental cellular functions such as proliferation, growth, and survival.

## Results

A total of 75 women were recruited for the purposes of this study. Eleven were excluded (low-quality ion images (n = 4) and tissue sections containing myometrium only (n = 7), leading to a final cohort of 64 eligible women (50 endometrial endometrioid cancer and 14 benign controls). Detailed patient characteristics are shown in Table 1. There were no statistically significant differences for the patient characteristics except for the menopause status. The mean age for controls was 54 y (SD: 12.1, range: 37 to 73) vs. 65 y (SD: 12.5, range: 29 to 90) for the cancer cases (*P* = 0.07). The rate of postmenopausal women was higher in the cancer when compared to the benign group (43/50, 86% vs. 8/14, 57%, *P* = 0.028). More women were Caucasian, nulliparous, nondiabetic, and of normal body mass index (BMI) in the benign group, although differences were nonsignificant. The majority of cancer cases were of early stage (stage I, 36/50, 72%). There was equal distribution of tumors across different grades.

**Table 1.** Patient characteristics

Participant characteristics	Endometrial cancer N = 50 n/N (%)	Benign controls N = 14 n/N (%)	Total N = 64 n/N (%)	*P* value
**Age (years)**				0.07
Mean (SD, range)	65 (12.5, 29 to 90)	54 (12.1, 37 to 73)	62 (13.1, 29 to 90)	
**Ethnicity, n/N (%)**				0.078
Caucasian	26/50 (52)	11/14 (79)	37/64 (58)	
Black/Asian/Mixed ethnic groups	17/50 (34)	1/14 (7)	18/64 (28)	
Unknown	7/50 (14)	2/14 (14)	9/64 (14)	
**Parity, n/N (%)**				0.187
Nulliparous	14/50 (28)	7/14 (50)	21/64 (33)	
Parous	33/50 (66)	6/14 (43)	39/64 (61)	
Unknown	3/50 (6)	1/14 (7)	4/64 (6)	
**Menopause status, n/N (%)**				0.028
Premenopausal	7/50 (14)	6/14 (43)	13/64 (20)	
Postmenopausal	43/50 (86)	8/14 (57)	51/64 (80)	
**Smoking status, n/N (%)**				0.568
Non-smoker/Former smoker	46/50 (92)	14/14 (100)	60/64 (94)	
Current smoker	4/50 (8)	0/14	4/64 (6)	
**Family history, n/N (%)**				0.346
No family history of cancer, 1st degree relative	31/50 (62)	11/14 (79)	42/64 (66)	
Family history of cancer, 1st degree relative	19/50 (38)	3/14 (21)	22/64 (34)	
**BMI distribution, n/N (%)**				0.170
Normal (18.5 to 25.0)	11/50 (22)	6/14 (43)	17/64 (27)	
Overweight/Obese (>25.1)	39/50 (78)	8/14 (57)	47/64 (73)	
**Diabetes status, n/N (%)**				0.431
Non-diabetic	40/50 (80)	13/14 (93)	53/64 (83)	
Diabetic	10/50 (20)	1/14 (7)	11/64 (17)	
**Cancer stage, n/N (%)**				n/a
IA	20/50 (40)	—	—	
IB	16/50 (32)	—	—	
II	4/50 (8)	—	—	
III (A-B-C1-C2)	8/50 (16)	—	—	
IV (A-B)	2/50 (4)	—	—	
**Cancer grade, n/N (%)**				n/a
1	15/50 (30)	—	—	
2	19/50 (38)	—	—	
3	15/50 (30)	—	—	
Unknown	1/50 (2)	—	—	

### Discrimination of Endometrial Cancer vs. Benign Cases.

**Fig. 2.**
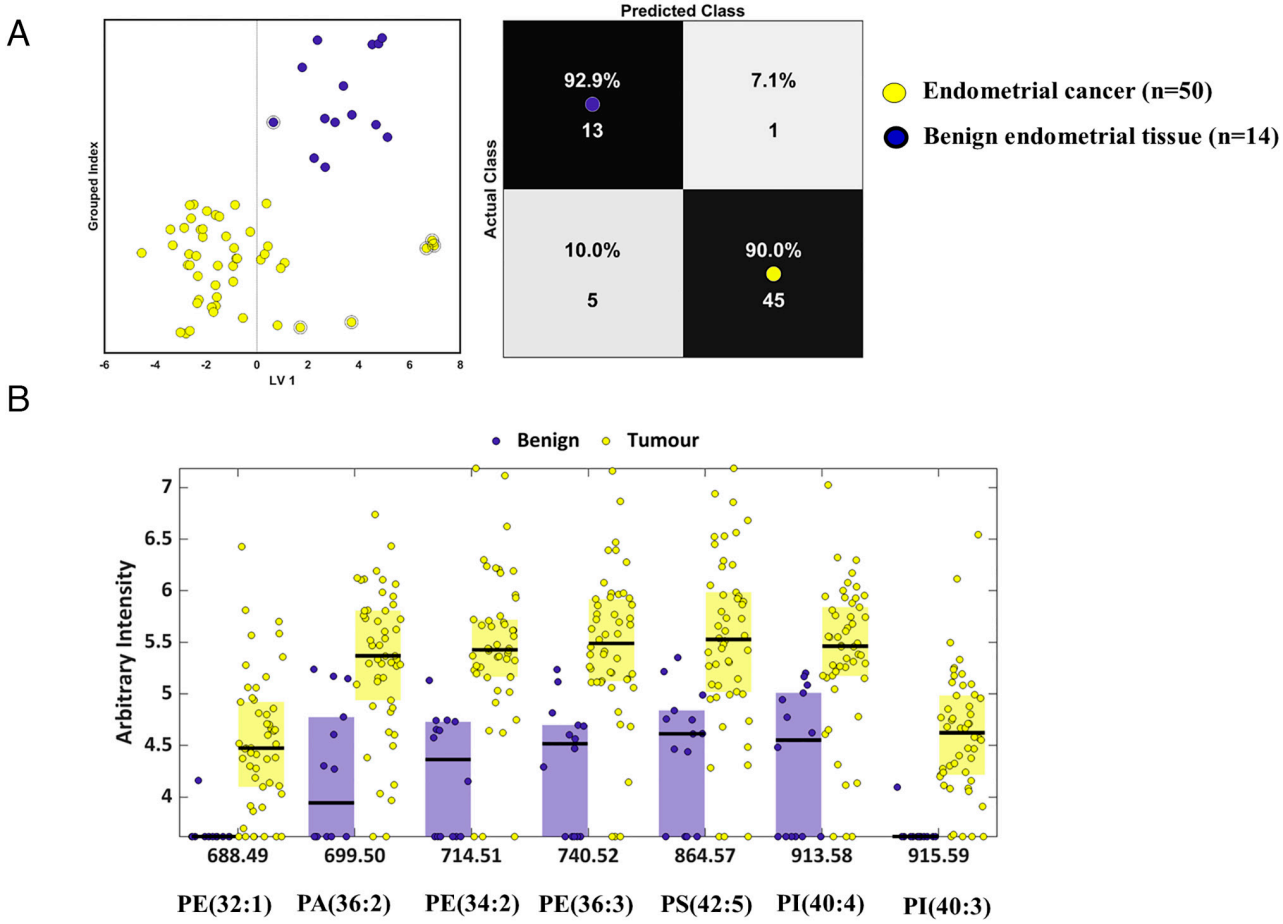
DESI-MSI analysis across all samples (normal vs. cancer). (*A*) Scores plot after recursive maximum margin criteria (RMMC) with each point representing a patient and confusion matrix after leave-one-out cross validation showing 90% sensitivity (true positive rate) and 93% specificity (true negative rate). (*B*) Discriminatory spectral features responsible for the observed differentiation between benign and cancer tissues (y-axis: log_10_-transformed).

We studied the lipidomic differences across the entire cohort; cancer (n = 50) vs. benign (n = 14). The regions of interest within each tissue section were considered as a class (i.e., normal or cancer) and multivariate approaches were further used to allow differentiation between classes. After supervised classification, the sensitivity and specificity for detecting endometrial cancer were 90% and 93% respectively (Fig. 2*A*). The discriminatory spectral features most responsible for this segregation between the groups were *m/z* 688.49, phosphatidylethanolamine (PE(32:1)); *m/z* 699.50, phosphatidic acid (PA(36:2)); *m/z* 714.51, PE(34:2); *m/z* 740.52, PE(36:3); *m/z* 864.57, phosphatidylserine (PS(42:5)); *m/z* 913.58, phosphatidylinositol (PI(40:4)); *m/z* 915.59, PI(40:3). Overall, the intensity of these peaks was higher in endometrial cancer in comparison to controls (Fig. 2*B* and Table 2).

**Table 2.** Identification of spectral peaks that were responsible for the observed differentiation between the different cohorts after univariate analysis

Subgroup comparison	Relative abundance	Input mass (m/z)	Theoretical mass (m/z)	Error (ppm)	Name	Adduct Ion	MS/MS	q value	Fold Change
Cancer vs. Benign	**↑** Tumor	688.4921	688.4923	−0.2905	PE 32:1	[M-H]^−^	PE (16:1_16:0)/PE (18:1_14:0)	5.7 × 10^−6^	1.24
	**↑** Tumor	699.5014	699.4970	6.2902	PA 36:2	[M-H]^−^	PA (18:1_18:1)	1.2 × 10^−6^	1.27
	**↑** Tumor	714.5123	714.5079	6.1581	PE 34:2	[M-H]^−^	PE (16:1_18:1)	6.8 × 10^−7^	1.28
	**↑** Tumor	740.5229	740.5236	−0.9453	PE 36:3	[M-H]^−^	PE (18:1_18:2)	1.4 × 10^−6^	1.27
	**↑** Tumor	864.5707	864.5760	−6.1302	PS 42:5	[M-H]^−^	PS (22:1_20:4)/PS (22:4_20:1)	4.1 × 10^−5^	1.24
	**↑** Tumor	913.5778	913.5812	−3.7216	PI 40:4	[M-H]^−^	PI (18:0_22:4)	1.1 × 10^−5^	1.23
	**↑** Tumor	915.5901	915.5968	−7.3176	PI 40:3	[M-H]^−^	PI (18:1_22:2)/PI (18:0_22:3)	1.8 × 10^−7^	1.27
High-risk vs. low-risk within benign cohort	**↑** High-risk phenotype	753.5636	753.5633	0.3981	^13^C isotope PE (O-38:4)	[M-H]^−^	^13^C isotope PE (O-18:0_20:4)/PE (O-18:1_20:3)	0.0241	4.03
	**↑** High-risk phenotype	813.5425	813.5481	−6.8834	^13^C isotope PS 38:3	[M-H]^−^	^13^C isotope PS (18:0_20:3)	0.0227	3.09
	**↑** High-risk phenotype	839.5628	839.5637	−1.0720	^13^C isotope PS 40:4	[M-H]^−^	^13^C isotope/PS (18:0_22:4)	0.0154	10.97
	**↑** High-risk phenotype	890.5829	890.5917	−9.8811	PS 44:6	[M-H]^−^	PS (22:4_22:2)	0.0309	5.06
High-risk vs. low-risk within cancer cohort	**↑** Low-risk phenotype	714.5123	714.5079	6.1581	PE 34:2	[M-H]^−^	PE (16:1_18:1)	0.0225	1.84
	**↑** Low-risk phenotype	740.5229	740.5236	−0.9453	PE 36:3	[M-H]^−^	PE (18:1_18:2)	0.0444	1.63
	**↑** Low-risk phenotype	753.5636	753.5633	0.3981	^13^C isotope PE (O-38:4)	[M-H]^−^	^13^C isotope PE (O-18:0_20:4)/PE (O-18:1_20:3)	0.0482	1.69

Tentative annotations were performed using database searches (LipidMaps, Metlin) and further confirmed using MS/MS analysis.

The sensitivity and specificity in the discrimination of cancer from benign were further improved in the subgroup analyses with more aggressive (grade) and advanced disease (stage). In the subgroups analyses by grade, the accuracy parameters slightly improved when compared grade 2 and 3 cancers vs. benign controls (sensitivity 93%, specificity 91%), grade 3 vs. benign (sensitivity 93%, specificity 87%), while this dropped for low grade disease (grade 1 vs. benign; sensitivity 86%, specificity 80%). The comparison of advanced stage vs. benign was further improved (stage 2 to 4 vs. benign; sensitivity 93%, specificity 93%), while this was decreased when comparing early disease (stage 1 vs. benign; sensitivity 86%, specificity 86%) (*SI Appendix*, Table S1).

The ability of the technology to discriminate between disease grades and stages was mediocre. DESI-MSI performed better when comparing moderate and high differentiation from low grade disease (grade 1 vs. grade 2/3 disease; sensitivity was 60% and specificity 82%—grade 1 vs. grade 2; sensitivity was 67% and specificity 79%—grade 1 vs. grade 3; sensitivity was 60% and specificity 67%) while performance was moderate in discriminating stage 1a vs. 1b or more (sensitivity 70%, specificity 55%) (*SI Appendix*, Table S1).

### Comparison of Lipidomic Profiles in High- and Low-Risk Phenotypes in Cancer and Benign Samples.

**Fig. 3.**
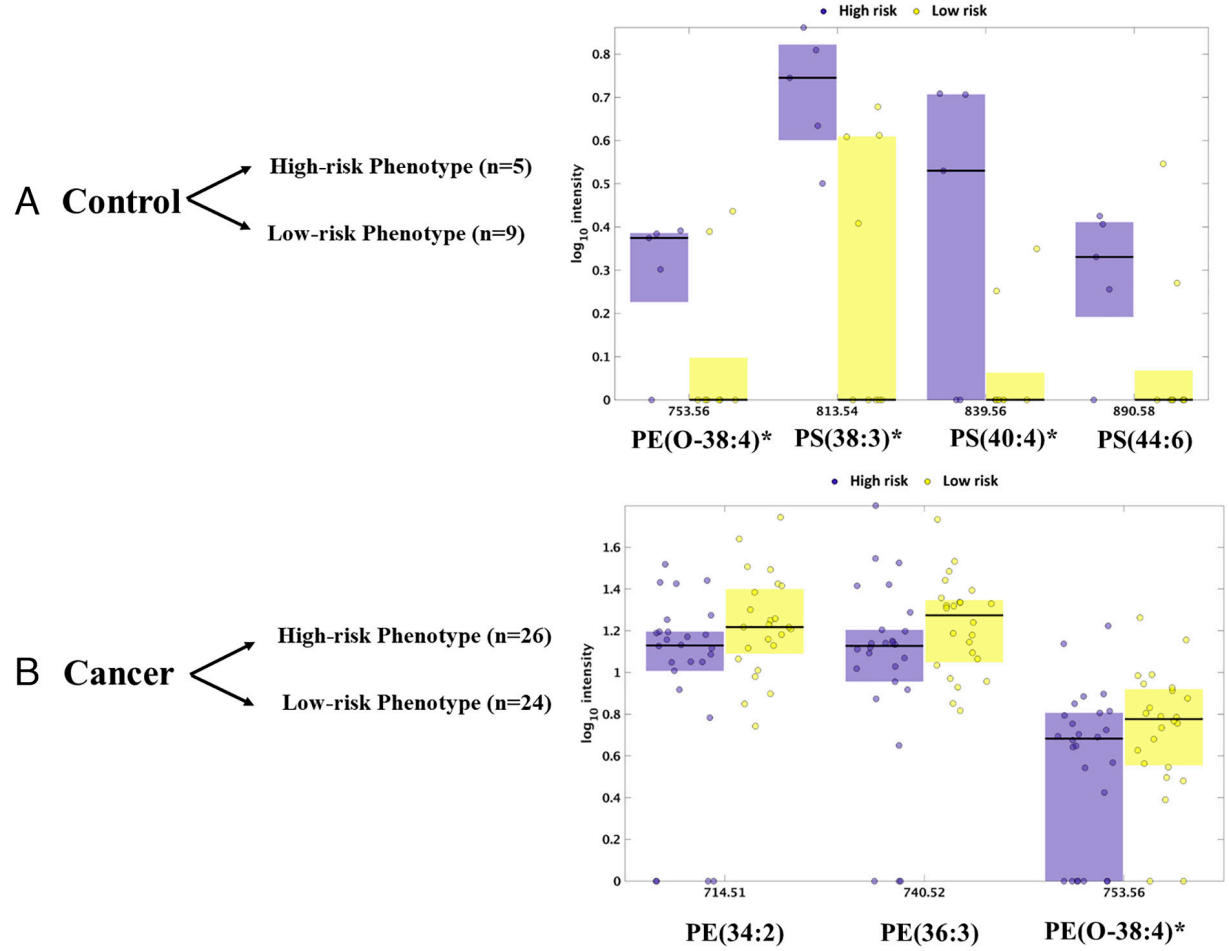
Investigation of the lipidomic profiles according to phenotype. (*A*) Discriminatory features between controls with a high-risk (obesity and/or diabetes) and low-risk (no obesity/diabetes) phenotype. (*B*) Discriminatory features between cancer samples with a high- and low-risk phenotype. *^13^C isotopes.

We explored the presence of spectral features that would discriminate between high- vs. low-risk phenotypes for cancer and benign samples separately (cancer = 50; low-risk 24, high-risk 26 – benign = 14; low-risk 9, high-risk 5). The spectral features that were found to be increased in the high-risk phenotype group in comparison to the low-risk in benign controls were *m/z* 753.56 ^13^C isotope of PE(O-38:4); *m/z* 813.54, ^13^C isotope of PS(38:3); *m/z* 839.56, ^13^C isotope of PS(40:4); *m/z* 890.58, PS(44:6) (Fig. 3*A* and Table 2 and *SI Appendix*, Fig. S2). In the cancer cohort, the low-risk phenotype group had increased intensity when compared to high-risk for the following discriminatory peaks: *m/z* 714.51, PE(34:2); *m/z* 740.52, PE(36:3); *m/z* 753.56, ^13^C isotope of PE(O-38:4) (Fig. 3*B* and Table 2 and *SI Appendix*, Fig. S2). PE(O-38:4) was found as discriminatory in both normal and cancer cohorts, which might serve as a biomarker to detect women with changes in tissue who are at increased risk of developing endometrial cancer in the future.

### Proteomic Analysis Using RPPA.

Proteomic data were obtained from 62/64 (97%) women included in the total cohort. This exploratory study identified a number of candidate proteins with altered expression between endometrioid endometrial cancer and benign endometrial tissues. Overall, 104 proteins were differentially expressed in cancer vs. benign cases (FDR q-value < 0.05). Commonly dysregulated pathways in endometrioid endometrial cancer, namely the PI3K/AKT/mTOR, MAPK/RAS, Wnt, and PLD signaling pathways, were further investigated to identify the protein expression levels in cancer and control cases. Twenty-three proteins implicated in these pathways were found to be significantly dysregulated between the two cohorts, with five being increased and 18 decreased in the cancer cohort (Table 3).

**Table 3.** Characteristics of protein expression after RPPA analysis according to histology type: endometrioid endometrial cancer (all grades) or benign

Signaling pathway	Antibody name	Phosphorylation site	Gene name	Validation status	Cancer mean	Cancer SD	Benign mean	Benign SD	FDR (q-value)	Dysregulation in cancer
**PI3K/AKT/mTOR pathway**										
GFR/EGFR	**EGFR_pY1173**	Y1173	EGFR	Validated antibody for RPPA	0.9421	0.1128	1.1650	0.1992	0.0086	Decreased
BAD/BCL2L8	**BCL2A1**		BCL2A1	Validated antibody for RPPA	0.9849	0.1898	1.2489	0.2565	0.0216	Decreased
4EBPs/EIF4EBP1/4E-BP1	**4E-BP1_pS65**	S65	EIF4EBP1	Validated antibody for RPPA	0.8778	0.3409	1.2403	0.5734	0.0392	Decreased
R/IGF1R	**IGF1R_pY1135_Y1136**	Y1135 Y1136	IGF1R	Validated antibody for RPPA	0.9314	0.1317	1.2889	0.1853	0.0086	Decreased
mTOR	**mTOR**		MTOR	Validated antibody for RPPA	1.1419	0.2033	0.7911	0.2286	0.0089	Increased
Rictor	**Rictor_pT1135 Rictor**	T1135	RICTOR RICTOR	Validated antibody for RPPA Validation in progress	0.9422 1.9480	0.1349 2.9653	1.1881 5.8460	0.1604 4.4807	0.0086 0.0147	Decreased Decreased
**MAPK/RAS pathway**										
RAF/ARAF/RAF1-CRAF/BRAF	**B-Raf_pS445 C_Raf_pS338**	S445 S338	BRAF RAF1	Validated antibody for RPPA Validated antibody for RPPA	1.0996 0.9325	0.3096 0.1069	0.7366 1.1221	0.1921 0.1052	0.0114 0.0086	Increased Decreased
ERK/MAPK1/MAPK3	**MAPK_pT202_Y204**	T202 Y204	MAPK3	Validated antibody for RPPA	0.9538	0.2218	1.4142	0.4277	0.0114	Decreased
Elk-1	**Elk1_pS383**	S383	ELK1	Validation in progress	0.8640	0.2501	1.3878	0.4742	0.0086	Decreased
Tau	**Tau**		MAPT	Validation in progress	0.9750	0.2741	1.3889	0.5491	0.0211	Decreased
MNK1/2	**MNK1**		MKNK1	Validated antibody for RPPA	1.1876	0.3382	0.7497	0.2706	0.0086	Increased
CREB	**Creb**		CREB1	Validation in progress	0.9150	0.1419	1.2435	0.3608	0.0153	Decreased
C-Myc	**C-Myc**		MYC	Validation in progress	0.8549	0.2705	1.5005	0.6986	0.0167	Decreased
JNK	**JNK_pT183_Y185**	T183 Y185	MAPK8	Validated antibody for RPPA	0.9466	0.1083	1.1666	0.1956	0.0086	Decreased
API/FOS/JUN	**c-Jun_pS73**	S73	JUN	Validated antibody for RPPA	0.9598	0.1457	1.1929	0.2775	0.0172	Decreased
PAK1/2	**PAK1**		PAK1	Validated antibody for RPPA	1.3264	0.8387	0.5019	0.2250	0.0086	Increased
ETS/ETS-1	**Ets-1**		ETS1	Validated antibody for RPPA	0.9303	0.1609	1.1898	0.2669	0.0114	Decreased
**Wnt pathway**										
B catenin	**b-catenin**		CTNNB1	Validated antibody for RPPA	1.1260	0.3734	0.6331	0.2519	0.0086	Increased
**PLD pathway**										
PLCγ	**PLC-gamma2_pY759**	Y759	PLCG2	Validation in progress	0.9826	0.1019	1.1648	0.1186	0.0086	Decreased
PKCα	**PKCa**		PRKCA	Validated antibody for RPPA	1.1321	0.7680	1.9067	0.9954	0.0371	Decreased
SHP2	**SHP-2_pY542**	Y542	PTPN11	Validation in progress	0.9227	0.1833	1.7356	0.7135	0.0114	Decreased

More specifically, within the PI3K/Akt/mTOR pathway, the following pathways and proteins were downregulated in cancer: GFR/EGFR (EGFR_pY1173), BAD/BCL2L8 (BCL2A1), 4E-BP1 (4E-BP1_pS65), IGF1R (IGF1R_pY1135_Y1136), Rictor (Rictor_pT1135 and Rictor), whereas mTOR was upregulated in the cancer cohort. In the MAPK/Ras pathways, nine proteins were downregulated including C-Raf_pS338, MAPK_pT202_Y204, Elk1_pS383, Tau, Creb, C-Myc, JNK_pT183_Y185, c-jun_pS73, and Ets_1, while three proteins were upregulated in cancer including B-Raf_pS445, MNK1, and PAK1. B-catenin, which is a key component of the Wnt signaling pathway, was also found upregulated in the cancer cohort. Three more pathways and proteins from the PLD pathway were found downregulated in cancer, including PLCγ (PLC-gamma2_pY759), PKCα, SHP2 (SHP-2_Py542).

### Correlation Analysis of Key Lipidomic and Proteomic Data.

**Fig. 4.**
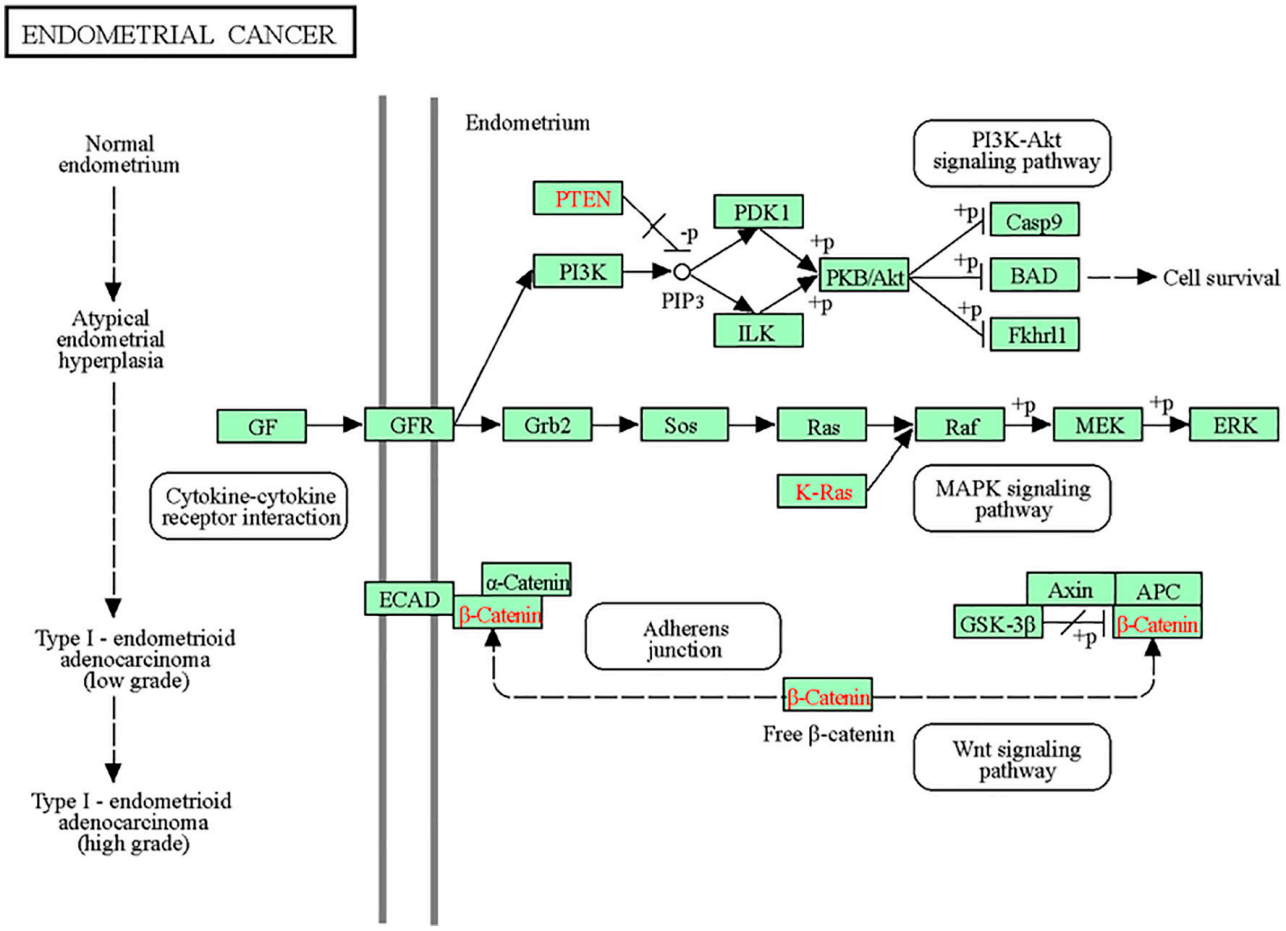
Commonly dysregulated pathways in endometrioid endometrial cancer (Type I), namely PI3K/AKT/mTOR, MAPK/RAS, and Wnt signaling pathways. Figure reproduced from KEGG Pathway Database.

**Fig. 5.**
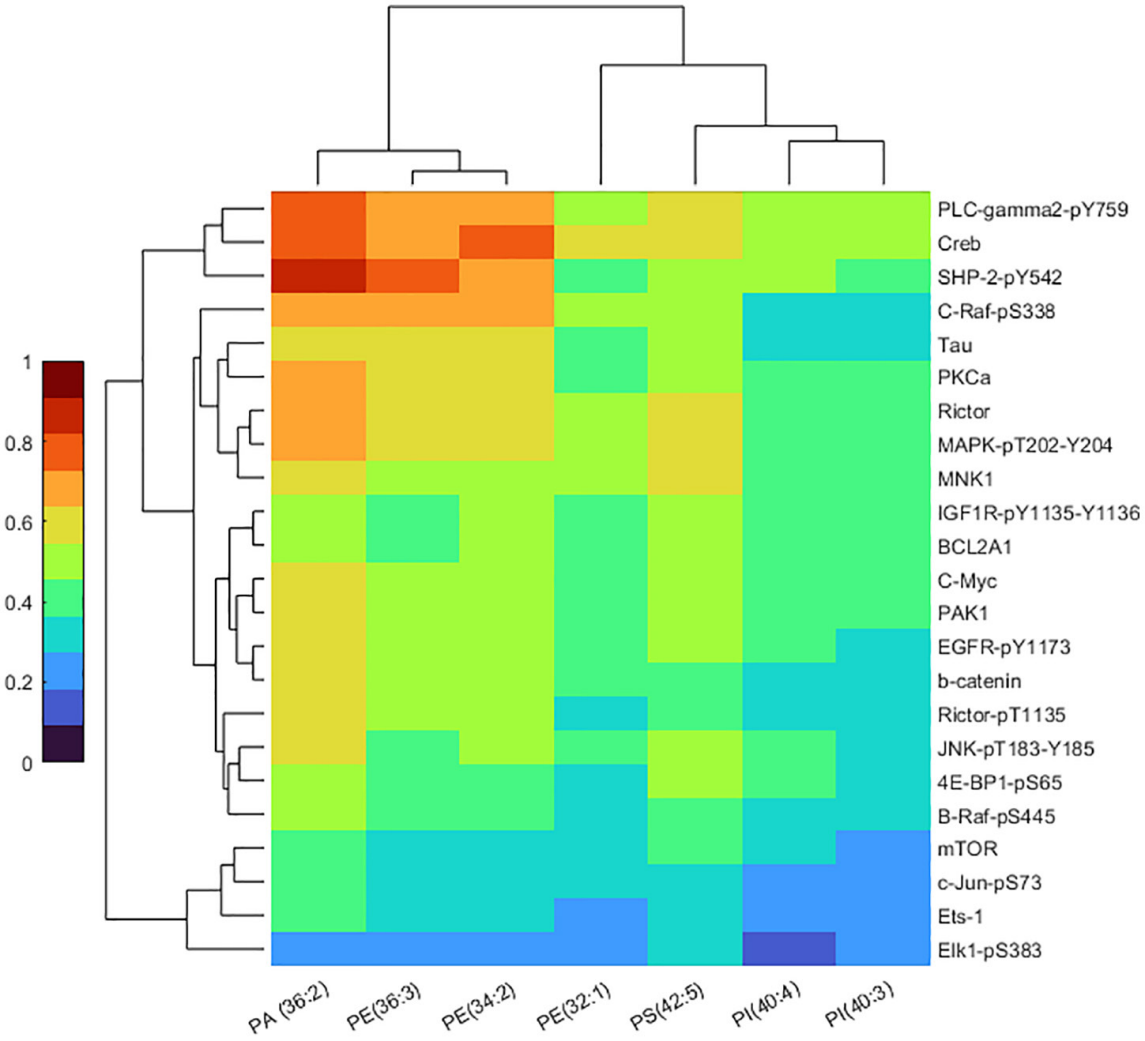
Heatmap after correlation analysis of the key lipidomic and proteomic features that were responsible for the differentiation between the benign and cancer cases. Heatmap was obtained after performing canonical correlations analysis with cosine distance metrics. The interprotein and interlipid clustering patterns are clearly depicted and correlation strengths are indicated by the color key.

Multimodal correlation of the seven lipids that were increased in the cancer cohort with the 23 dysregulated proteins were performed by canonical correlation analysis with a cosine distance metric group. The latter group includes proteins that have been implicated in commonly dysregulated endometrioid endometrial cancer pathways, namely the PI3K/AKT/mTOR, MAPK/RAS, Wnt (Fig. 4), and PLD signaling pathways. A cluster heatmap was generated to visualize the relationship between specific lipids and proteins within the same cohort of women (Fig. 5). A strong positive correlation was observed between PA(36:2), PE(34:2), PE(36:3), PS(42:5) and the proteins C-Raf_pS338, Creb, PLC-gamma2_pY759, and SHP-2_Py542. High correlation was also observed between PA(36:2) and the PKCa, Rictor, and MAPK_pT202_Y204 proteins.

## Discussion

Although diagnostic tools for pre- and postoperative management of endometrial cancer rely predominantly on histopathological diagnosis, innovative technologies like DESI-MSI have the potential to enable an objective, user-independent, rapid, label-free diagnosis while providing insight into the pathophysiology of the disease in a single analysis. This study describes the application of DESI-MSI in the investigation of endometrial cancer.

In this study, we used DESI-MSI to explore the lipidomic profile of benign and malignant endometrial samples with the primary objective to assess its diagnostic accuracy. The technology was able to discriminate cancer from benign samples with high accuracy and holds promise as an adjunctive diagnostic tool in pathology laboratories permitting faster, automated diagnosis. DESI-MSI also holds potential as a tool for intraoperative assessment of surgical margins and differential diagnosis of endometrial cancer subtypes, which would allow instant management decisions and improve patient outcomes (32). The technique discriminated endometrioid endometrial cancer from benign samples with a sensitivity and specificity of 90% and 93%, respectively. Discrimination of cancer from benign was further improved in the subgroup analyses with more aggressive (grade) and advanced disease (stage), however, the diagnostic performance of the technology in discriminating between grades of differentiation and early- from late-stage cancers was mediocre but the number of samples in each group was small. Following validation of these results in an independent larger cohort, this technology could be used to enhance clinical pathways by investigating abnormal uterine bleeding and assisting histopathological diagnosis by prioritizing samples at high risk of malignancy while it could also develop into a tool for intraoperative analysis.

The observed differentiation between cancer and benign samples was attributed to higher relative abundance of phospholipids, including PE, PA, PS, and PI containing polyunsaturated fatty acyl chains, all of which have been previously implicated in carcinogenesis (20, 27, 33–35). Numerous studies have reported a link between increased phospholipid levels, carcinogenesis, and cancer metastasis (36). The high demand of energy needed during malignant transformation and accelerated cancer cell proliferation induces alterations in lipid metabolism in order to allow the survival of cancer cells (36). In a study using liquid chromatography coupled with high-resolution mass spectrometry (LC-HRMS), PE levels were also significantly increased in endometrial cancer tissues (n = 34) in comparison to normal tissues (n = 34) (37), which is in line with our findings. Trousil et al. (38) also reported that the lipid metabolism was found severely dysregulated in endometrial cancer with phosphocholine in particular being 70% higher in cancerous tissue when compared to normal tissue samples.

More recently, Feider et al. explored the role of DESI-MSI in the detection of endometriosis (21), a known precursor for endometriosis-related carcinomas (39–41), with promising results, which were in line with other studies reporting dysregulation of lipid metabolism (39) and other biochemical pathways in endometriosis (40, 42). Specifically, DESI-MSI detected endometriosis lesions with high accuracy and identified molecular markers that were significantly altered in ectopic endometrial tissues when compared to eutopic tissues, including fatty acids and phospholipids (PS) (21). Li et al. used ultra-high performance liquid chromatography coupled with electrospray ionization high-resolution mass spectrometry (UHPLC-ESI-HRMS) and achieved 91% sensitivity and 75% specificity in distinguishing women with endometriosis and controls (43) with lipid alterations, such as decreased concentrations of PC and PS and increased PA, detected at an early stage of endometriosis (Stage I to II).

DESI-MSI is an innovative technology providing molecular classification that may offer further insight into disease adjuvant treatment, aggressiveness, prognosis, and survival. A study conducted by The Cancer Genome Atlas (TCGA) Research Network presented an in-depth genome-wide analysis of endometrial cancers from 373 women and described four distinct types of tumors according to their genomic patterns: *POLE* ultramutated, microsatellite instability hypermutated, copy-number low, and copy-number high (44). The authors proposed a reclassification of endometrial tumors, which might have clinical relevance for postsurgical adjuvant treatment of aggressive tumors. DESI-MSI allows the investigation of small metabolites and complex phospholipids and can detect dysregulated lipid metabolism caused by carcinogenesis. Future follow-on studies should explore whether DESI-MSI could discriminate with accuracy among these four tumor subtypes.

The high diagnostic performance of DESI-MSI is in line with accuracies achieved by histopathological examination. More specifically, in a study of 360 endometrial cancer cases the sensitivities of pipelle biopsy and curettage were 94 and 97% in low-grade cancer and 99 and 100% in high-grade cancer (45). In a meta-analysis including 34 studies (7,914 women) to assess the diagnostic performance of pipelle biopsy, dilation and curettage and hysteroscopy in endometrial cancer, the pipelle biopsy provided the best diagnostic accuracy with detection rates of 100% and 91% for postmenopausal and premenopausal women, respectively (46). These findings determine the need for a complementary more objective and accurate diagnostic test, such as the DESI-MSI test proposed in this study. Moreover, the depth of myometrial invasion in endometrial tissue sections determines histopathological staging and is used to select adjuvant treatment with radiation and/or chemotherapy. This may be of further value in the assessment of surgical margins and lymph node involvement. Advanced technologies, such as DESI-MSI, have the potential to enhance histopathological diagnosis in the laboratory.

**Fig. 6.**
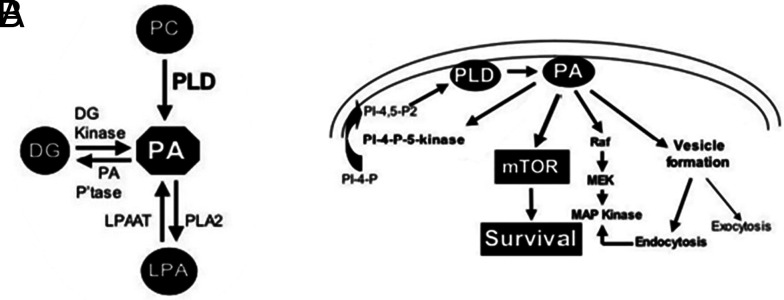
(*A*) Phosphatidic acid (PA) is generated primarily through the hydrolysis of phosphatidylcholine (PC) by PLD but can also be generated through the phosphorylation of diacylglycerol (DG) and from lysophosphatidic acid (LPA) via an acylation reaction. (*B*) PA and its downstream targets. PA activates phosphatidylinositol-4-phosphate (PI-4-P)-5-kinase, which may create a positive feedback loop to generate the PI-4,5-bis-phosphate (PI-4,5-P2) required for PLD activity. The likely relevant targets for survival signals provided by PLD and PA are Raf and mTOR, which regulate progression through the cell cycle and suppress apoptosis. PA is also required for endocytosis, which along with Raf, contributes to the activation of MAP kinase. Figure reproduced from Foster et al. (53).

The secondary objective of this study was to explore the ability of the technology to detect a lipidomic profile that could detect individuals at high risk of developing cancer. To date, there are no interventions shown to be of value in prevention of endometrial cancer. A common spectral feature, attributed to PE(O-38:4), was identified as discriminatory in both cancer and benign cohorts after comparison of women with a high-risk (obesity and/or diabetes) and low-risk (no obesity/diabetes) phenotype. PE(O-38:4) is a plasmalogen, a subclass of glycerophospholipids which have been associated with diverse clinical manifestations including metabolic diseases associated with oxidative stress (47) and cancer (48). Previous studies suggest that changes in plasmalogen metabolism may contribute to the development of various cancer types (49), while they have also been correlated with spreading metastasis (50, 51). A previous DESI-MSI study also reported elevated concentration of PE plasmalogen species after comparing cancerous and healthy tissues (24). Therefore, this may serve as a biomarker to detect women with benign histology who are at increased risk of developing endometrial cancer in the future, however the ability to predict which individuals at high-risk will develop cancer remains uncertain and requires prediagnostic samples in longitudinal cohorts. Preinvasive precursors, such as hyperplasia and atypia, have been previously described (14, 52) but the predictive values of more subtle changes, like increased or disordered proliferation, remains unclear. Nevertheless, previous studies have reported severe dysregulation of the lipid metabolism in both endometrial cancer and atypical hyperplasia cases when compared with normal endometrial tissues, with increased phosphocholine levels being the most important lipid-related alterations (38). Phosphatidylcholine (PC), synthesised from phosphocholine via the Kennedy pathway, is hydrolysed by phospholipase D (PLD) to primarily generate phosphatidic acid (PA) (Fig. 6*A*), and therefore overexpression of PC may subsequently result in the overexpression of PA and its conversion to PE, PS, PI, and PG. Overall, after comparing the lipidomic profiles of low- vs. high-risk individuals with obesity and/or diabetes, we identified elevated levels of phospholipid species, such as PE and PS in the benign high-risk cohort, whereas women with cancer presented with decreased levels of PE in the high-risk group.

In endometrial cancer, PLD expression is controlled by a combination of mechanisms, including the activation of key signaling pathways. More specifically, activation of the PI3K/Akt pathway, which is often upregulated in endometrial cancer (54) (Fig. 4), can lead to increased PLD expression (*SI Appendix*, Fig. S1). This pathway influences cell growth and survival and can upregulate PLD as part of its oncogenic effects. Similarly, the MAPK and Wnt pathways, which are also commonly dysregulated in endometrial cancer (55, 56) (Fig. 4 and *SI Appendix*, Fig. S1), can enhance PLD expression through their downstream effects on transcription factors and other regulatory proteins. These mechanisms collectively contribute to the upregulation of PLD, which can promote cancer cell proliferation, survival, and metastasis. Stimulating the activation of PLD leads to the upregulated production of PA, which is the precursor phospholipid for membrane biosynthesis and can be converted to PE, PS, PI, and PG, with multiple functions that regulate key physiological and pathological cell processes (cell growth, differentiation, apoptosis) and stimulates the mammalian target of rapamycin (mTOR) pathway, promoting cell proliferation (Fig. 6*B*) (57). Using high-resolution LC–MS and tandem MS, a study of 39 human endometrial cancer tissues and 17 healthy controls characterized the metabolomic and lipidomic profile of postmenopausal women (35). Significantly altered lipid pathways were observed in the cancer cohort with an important number of lipid species including PC, PS, PE, PI, PA, and PC being dysregulated, confirming our results.

The increased levels of phospholipids reported in this study in high-risk benign women may be caused by the presence of obesity and diabetes in this cohort and may be indicative of women at an increased risk of developing cancer. From a recent proteomics study in the same cohort of women (58), benign endometrial tissue in women with obesity and insulin resistance, which were considered to be “at-risk” of developing endometrial cancer, was found to have specific upregulation of proteins involved in insulin signaling and oncogenic signaling pathways. Numerous studies have also previously demonstrated a dysregulated lipid metabolism caused by metabolic syndromes, such as obesity and diabetes, which are the main risk factors for endometrial cancer development. Although the interrelationship between lipid metabolism, metabolic syndrome, and cancer has been established, the underlying biological mechanisms are not completely understood. Dyslipidemia with increased levels of lipids and lipoproteins has been associated with diseases such as obesity and diabetes (59). A mass spectrometry-based study aiming to identify the lipidomic biomarkers associated with obesity and insulin resistance also revealed significant associations with lipid concentrations (60).

DESI-MSI found increased levels of phospholipids in cancer cases, which were also supported by our RPPA results that showed overexpression of certain proteins (mTOR, B-Raf_pS445, MNK1, PAK1, b-catenin,) that are implicated in dysregulated endometrial cancer signaling pathways, such as the PI3K/Akt/mTOR, MAPK/RAS and Wnt pathways (Fig. 4). The PI3K/Akt/mTOR pathway has a critical role in the malignant transformation of human tumors and their subsequent survival, growth, proliferation, and metastasis (61). This pathway is overactivated in endometrial cancers and is directly linked to the phospholipid metabolism and PA pathways. Along other downstream targets, the PA class also has a direct role in the activation of the PI3K/Akt/mTOR signaling pathway by stimulating mTOR expression after competing with the action of rapamycin (Fig. 6*B*) (53, 62, 63).

Within the MAPK/RAS signaling pathway, B-raf_pS445, MNK1, and PAK1 were found to be increased in cancer following RPPA analysis. B-raf is a member of the Raf kinase family of growth signal transduction protein kinases and has a role in regulating the MAPK/ERK pathway (Fig. 4), which affects cell division, differentiation, and secretion. Apart from mTOR, Raf, which is dysregulated in cancer, is also a likely relevant target for survival signals provided by PLD and PA (Fig. 6*B*). MNK kinases play important roles as key mediators of oncogenic progression, production of proinflammatory cytokines and cytokine signaling. The upregulation of MNK1 (i.e., MAP kinase-interacting serine/threonine kinase 1) in cancer cases may be a downstream result from the upregulation of B-Raf (64). Increased levels of PA and PI in the cancer cohort (evidenced by DESI) also have stimulatory effects on PAK1 activity through the direct binding of PLD-derived PA to PAK1 or through the involvement of PI lipids in signaling pathways that activate PAK1 (65–67). Activated PAK1 participates in multiple cellular processes, including cytoskeletal reorganization, cell survival, and proliferation. Overexpression of PAK1 promotes endometrial cancer progression and cell proliferation (68) and previous studies have demonstrated that it is overexpressed in clinical samples of endometrial cancer when compared with normal endometrium (55).

Within the Wnt signaling pathway, b-catenin was also found to be increased in cancers in comparison to benign cases. B-catenin is a growth and proliferation-promoting protein and has been implicated in carcinogenesis, including the development of endometrial and ovarian endometrioid carcinomas (Fig. 4) (56). Previous findings demonstrated that the majority of endometrioid adenocarcinomas showed strong b-catenin expression, which is in line with our results (56). Other studies have also reported the emerging importance of PLD and PA in the Wnt/b-catenin signaling network, which is associated with tumorigenesis, showing that PLD and PA can lead to upregulation of b-catenin; for this reason, PLD/PA pathway has also been previously suggested as a potential therapeutic target for cancer (69, 70). Previous studies have demonstrated that PLD is a downstream target of proteins involved in inflammation and carcinogenesis. Consequently, compounds that inhibit PLD expression or activity could be beneficial in reducing inflammation and sensitizing resistant cancers during chemotherapy (71).

### Strengths and Limitations.

Lipidomics is a powerful analytical approach for studying lipid composition and metabolism within biological systems and can offer valuable insights into metabolic reprogramming under different conditions, such as disease states or response to specific treatments. Lipidomic techniques can allow the analysis of samples capturing dynamic changes in lipid levels/species during biological processes and provide information about the lipid metabolism and involved signaling pathways. Different mass spectrometry-based approaches, such as SIMS and MALDI, can be employed for spatially resolved analysis of tissues, however, these require complex sample preparation steps which are time consuming and can also introduce unwanted biases and variability. DESI-MSI is a sensitive, matrix-free technique that can allow imaging analysis of tissue samples in their native state after minimal sample preparation, which can simplify the workflow and reduce potential sample artifacts. Compared to other mass spectrometry techniques, DESI-MSI can offer faster imaging speeds and rapid data acquisition, making it suitable for large-scale imaging studies and high-throughput analyses. Operating under ambient conditions without the requirement for vacuum or cryogenic conditions, DESI-MSI also holds immediate translational potential as an intraoperative tool for the real-time analysis of tissues which would ensure surgical margin clearance and allow immediate management decisions during surgery, thus improving patient outcomes.

While other cancerous tissues, such as lung (20), breast (28–30), gastric (25), ovarian (27), and esophageal (31), have been previously successfully studied with DESI-MSI, this study investigates endometrial samples and characterizes their lipidomic profile. Our results using DESI-MSI show potential for an automated, objective, and rapid technique for the diagnosis of endometrioid endometrial cancer. Proteomics analysis was also conducted in the same cohort of women for further insight into the dysregulated signaling pathways, which correlated with the lipidomics findings. Further studies should validate the findings and assess the diagnostic accuracy of the technology when compared to existing pathology techniques. Although this technology may not replace existing histopathological diagnosis, it has the potential to offer an automated rapid tool that can enhance the diagnostic accuracy as an adjunctive tool. Further studies should aim to assess the technology’s accuracy to discriminate distinct clinical groups based on existing stage and grade cut-offs that determine prognosis and adjuvant treatments. We attempted further exploration of the lipidomic signature in high- and low-risk subgroups in women with cancer and those without. Although common features were present, we were limited by the small numbers in each group. While the overall cohort size (n = 74) compares favorably to other proof-of-concept studies in the field (72) and provides sufficient power to support our preliminary conclusions, the unequal distribution between cancer cases and controls represents a limitation that should be addressed in future validation studies. Nevertheless, within our subgroup analyses, group sizes were appropriately matched (e.g., 26 high-risk cancers vs. 24 low-risk cancers), demonstrating balanced comparisons where clinically relevant. As this represents the first application of DESI-MSI to endometrial cancer, these numbers are sufficient given the technical and practical challenges inherent in generating such datasets. Moreover, obese and/or diabetic individuals were grouped together although untangling the contribution of hyperestrogenemia and metabolic dysregulation may be difficult unless these groups are explored separately in larger clinical groups.

## Conclusion

DESI-MSI has the potential to discriminate samples with cancer from those with no disease with high diagnostic accuracy providing an objective automated adjunctive diagnostic tool in the histopathological laboratories. Phospholipid relative abundancies were higher in the cancer cohort when compared to controls, which confirms the well-documented lipid dysregulation that occurs during carcinogenesis. The technology may provide further insight into the pathophysiology and molecular pathways involved in endometrial cancer, which may subsequently lead to the identification of novel disease markers. Future larger cohorts should explore the diagnostic accuracy of the technology and further explore whether this has the potential to detect with accuracy the four molecular subtypes of the disease as identified by the TCGA Program. Future follow-on studies should further explore whether molecular lipidomic signatures in benign prediagnostic samples could predict high-risk individuals in longitudinal cohorts.

## Materials and Methods

### Study Population.

We recruited women undergoing hysterectomy for endometrial cancer and a control population having surgery for other benign gynecological conditions at Imperial College Healthcare NHS Trust, between 2014 and 2016. Women were included in the study irrespective of ethnicity, parity, menopause and smoking status, family history of cancer, BMI, or diabetes status. Nonendometrioid or mixed endometrial cancers, different primary site tumors of the female genital tract and women with previous hysterectomy were excluded. Ethical approval was obtained from the NHS West of Scotland Research Ethics Service Committee (REC:14/WS/1098) and Imperial College London and Imperial College Healthcare NHS Trust Joint Research Compliance Office (No. 14HH2220 CSP, Ref: 154598). All patients provided written informed consent prior to their inclusion in the study and all methods were performed according to institutional and ethical guidelines.

### Tissue Sample Collection, Preparation, and DESI-MSI Analysis.

Hysterectomy specimens were immediately collected from the operating theater and taken to the on-site pathology laboratory; a representative tissue sample including endometrial epithelium and stroma was provided by the consultant histopathologist. After collection, samples were immediately stored at -80 °C and later cryosectioned at 10 μm thickness, mounted onto a glass slide and stored at −80 °C until further use. For spectrometric analysis, the slides containing the tissue sections were mounted onto a 2D-linear moving stage and allowed to thaw under nitrogen flow.

DESI-MSI was performed on endometrial tissue samples to investigate their lipidomic signatures (Fig. 1). Analysis was performed using a Xevo-G2-XS Q-ToF mass spectrometer (Waters Corporation, UK). Mass spectra were collected using nitrogen pressure of 6.0 bar, sprayer voltage of 4.5 kV, capillary voltage 50 V, and a solvent mixture of methanol and water in a ratio 95:5 with a flow rate of 0.75 μL/min. Experimental analysis was carried out in the negative ion mode over the 50 to 1,500 mass range at a spatial resolution of 100 μm and speed rate of 100 μm/s. Samples were analyzed in a random fashion so as to not run similar tissue types (e.g., all benign samples) consecutively to avoid selection bias.

After MSI analysis, tissue sections were stained with H&E for histopathological diagnosis confirmation and annotation. Samples were classified into benign endometrial tissue and endometrioid endometrial cancer. The researcher conducting experimental work was blinded to the final diagnosis during analysis.

### DESI-MSI Data Analysis and Feature annotation.

Raw mass spectrometry images were converted to imzML format (73) using imzML Converter (vs. 1.0.5) and imported into MATLAB (R2016a) in-house toolbox for preprocessing and further analysis (74). Each histological image was coregistered to the DESI-MS image to enable accurate annotation of the regions of interest. The spectral profiles within the *m/z* 600 to 1,000 were aligned using an in-house peak-matching procedure subject to a maximum peak shift of 8 ppm, as described previously (27). Mass spectra from the same tissue section were averaged and normalized using mean probabilistic quotient normalization (PQN-mean) to correct for differences in mass spectral intensities.

We assessed whether differences in spectral signatures could discriminate between endometrioid endometrial cancer and benign endometrial samples. Recursive maximum margin criterion linear discriminant analysis (RMMC-LDA) using leave-one-patient-out (LOPO) cross-validation was used for supervised classification to define the diagnostic accuracy, sensitivity, and specificity. RMMC-LOPO cross validation evaluates the performance of the RMMC-LDA algorithm, which uses a maximum margin criterion to identify a linear boundary that separates two or more classes in the dataset. The algorithm iteratively identifies the sample with the least margin (i.e., the sample closest to the boundary) and removes it from the analysis. This process is repeated until all samples have been removed, resulting in a set of discriminant functions that can be used to classify new data. In RMMC-LOPO cross-validation, the original dataset is repeatedly sampled to create multiple training and validation sets. Each training set consists of a subset of the original data, while the validation set consists of a single sample that was left out of the training set. For each iteration of the cross-validation procedure, the RMMC-LDA algorithm is applied to the training set to identify the discriminant functions, which are then used to classify the validation sample. This process is repeated multiple times with different subsets of samples for training and validation each time. Following supervised analysis, a confusion matrix is obtained with the true positive (TP), true negative (TN), false positive (FP), and false negative (FN) values, from which the diagnostic performance of the technique can be assessed. ANOVA was employed as a univariate analysis approach to determine the discriminatory spectral features that were responsible for the differentiation between benign and cancer groups. ANOVA produces a *P* value which provides the probability of the difference between groups being due to chance alone, with a low *P* value of 0.05 suggesting that there is statistically significant difference. Since there are multiple univariate tests conducted, the likelihood of a false positive becomes significant and therefore q-values were computed by applying the false discovery rate (FDR) procedure to correct for multiple testing (75). Different subgroup analyses were also performed within the total cohort to assess the accuracy of the technique in discriminating cancers of varying grades and stages (ie. total cancer cohort) from benign controls as well as in correctly identifying the grades (G1 to G3) and stages (1 to 4) of cancer.

Further subgroup analysis was conducted to study the lipidomic profile of our cohort based on their phenotype. Women were grouped in different classes according to their histological diagnosis as benign controls or endometrial cancers and subsequently classed according to their phenotype as high- (obesity and/or diabetes) or low-risk (no obesity/diabetes). Spectral features that were found as discriminatory were subjected to annotation based on accurate m/z values and isotopic patterns and candidate lipids were established via database searches [LipidMaps (76), Metlin (77)]. Features of interest were further validated in situ in tissue using DESI MS/MS. In particular, discriminative m/z features, tentatively annotated primarily as glycerophospholipids (PA, PE, PI, and PS), were confirmed through DESI MS/MS analysis. The collision energy was manually optimized for each m/z value, typically within the range of 30 to 35 V. Lipid annotation was performed after manual inspection of the MS/MS spectra. Glycerophospholipid classes were assigned in negative ion mode by interpreting characteristic fatty acyl chain fragments and polar head group fragments. MS/MS annotations, along with input/theoretical masses and error (ppm) are provided for each feature in Table 2.

### Sample Preparation and Analysis for RPPA.

Each tissue sample was prepared for RPPA analysis based on previously described protocols (44, 78) and samples were sent to the RPPA Core Facility at MD Anderson Cancer Centre, USA, for processing and RPPA analysis (Fig. 1) (58). A total panel of 282 proteins were used for staining in RPPA, which have been described in detail in previous publication (58). A global primary assessment of protein expression profiles within the study cohort was conducted using unsupervised hierarchical clustering analysis of genes via centroid linkage using GeneCluster 3.0 (https://bonsai.hgc.jp/*mdehoon/software/cluster/software.htm) (58).

### Correlation Analysis of Key Lipidomic and Proteomic Data.

To integrate and interpret both lipidomic and proteomic data, we performed canonical correlation analysis on 7 upregulated lipids and 23 dysregulated proteins implicated in endometrial cancer pathways, namely the PI3K/AKT/mTOR, MAPK/RAS, Wnt, and PLD signaling pathways. Both datasets were first normalized by their total and maximum intensities, respectively, before analysis. The cosine distance between every possible pair was then computed to generate a heatmap visualizing the relative similarity between species. Unsupervised clustering analysis was also performed to visualize the grouping between lipid and protein species by means of dendrograms.

### Statistical Analysis.

We performed statistical analysis using IBM SPSS Statistics (version 26) to assess differences in patient characteristics. *P*-values were calculated using a *t* test for the age and a Fisher’s exact test for ethnicity, parity, menopause, smoking status, family history of cancer, BMI distribution, and diabetes status. A *P* value < 0.05 was considered significant.

The R statistical software (version 3.4.4) was used to examine the statistical associations in the RPPA data. A series of 282 univariate logistic regression models with a binomial link were used to relate each protein expression level to the clinical status of the tissue (benign vs. cancer). Odds ratios and their 95% CI intervals were calculated for all proteins (Dataset S1). To address the multiplicity issue, an FDR correction was applied, and q-values were computed with those below the threshold of 0.05 deemed statistically significant. The means and SD of each protein’s expression levels were provided both in benign and cancer groups. Linear regression analysis and independent t-tests were employed to examine proteomic expression in the cancer and benign tissues (58). Proteomic data from 560 endometrial cancer cases from The Cancer Genome Atlas (TCGA) databank were used to assess reproducibility of results (58).

## Supplementary Material

Appendix 01 (PDF)

Dataset S01 (XLSX)

## Data Availability

Mass spectral data are deposited in FigShare: DOI: https://doi.org/10.6084/m9.figshare.30553595.v1 (79).
