# Supplementary material for: Lipidomic profiling of endometrial cancer using desorption electrospray ionization mass spectrometry imaging
Source: Proc Natl Acad Sci U S A. 2025 Nov 24;122(48):e2522839122. doi: 10.1073/pnas.2522839122 (PMC12685106; doi:10.1073/pnas.2522839122)
Supplement: Supplementary file 1 — Appendix 01 (PDF) [file pnas.2522839122.sapp.pdf]

Supplementary Information

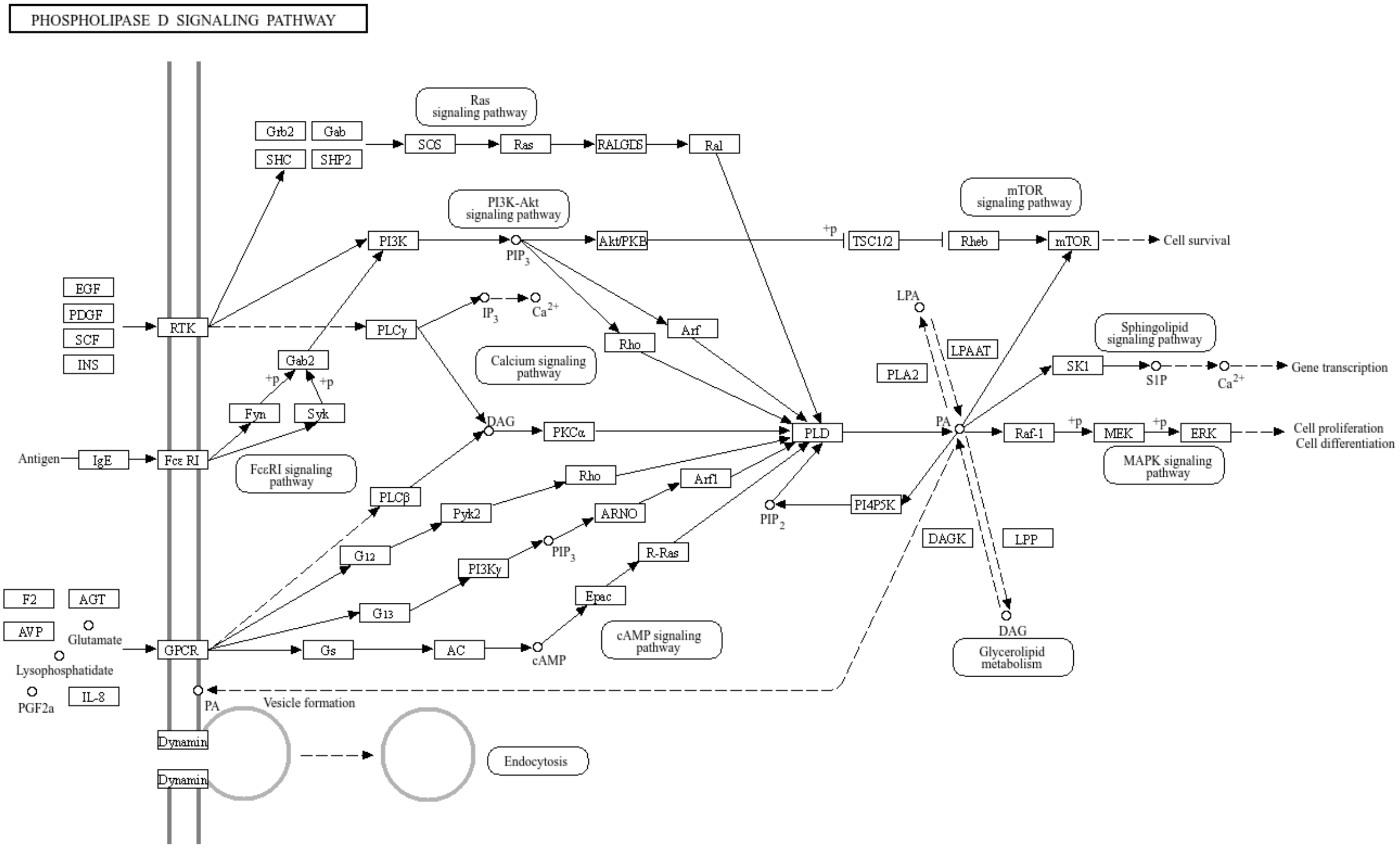

Supplementary Figure 1: Phospholipase D (PLD) signalling pathway. Growth factors (e.g., PDGF, EGF, insulin) bind to their respective receptors on the cell surface, causing receptor activation and initiating intracellular signaling cascades. Activation of key signalling pathways, such as the PI3K/Akt, MAPK/RAS and Wnt pathways, which are commonly dysregulated in endometrial cancer, contribute to the upregulation of PLD, which can promote cancer cell proliferation, survival and metastasis. Stimulating the activation of PLD leads to the upregulated production of phosphatidic acid (PA) and choline from phosphatidylcholine (PC). Figure reproduced from KEGG Pathway Database.

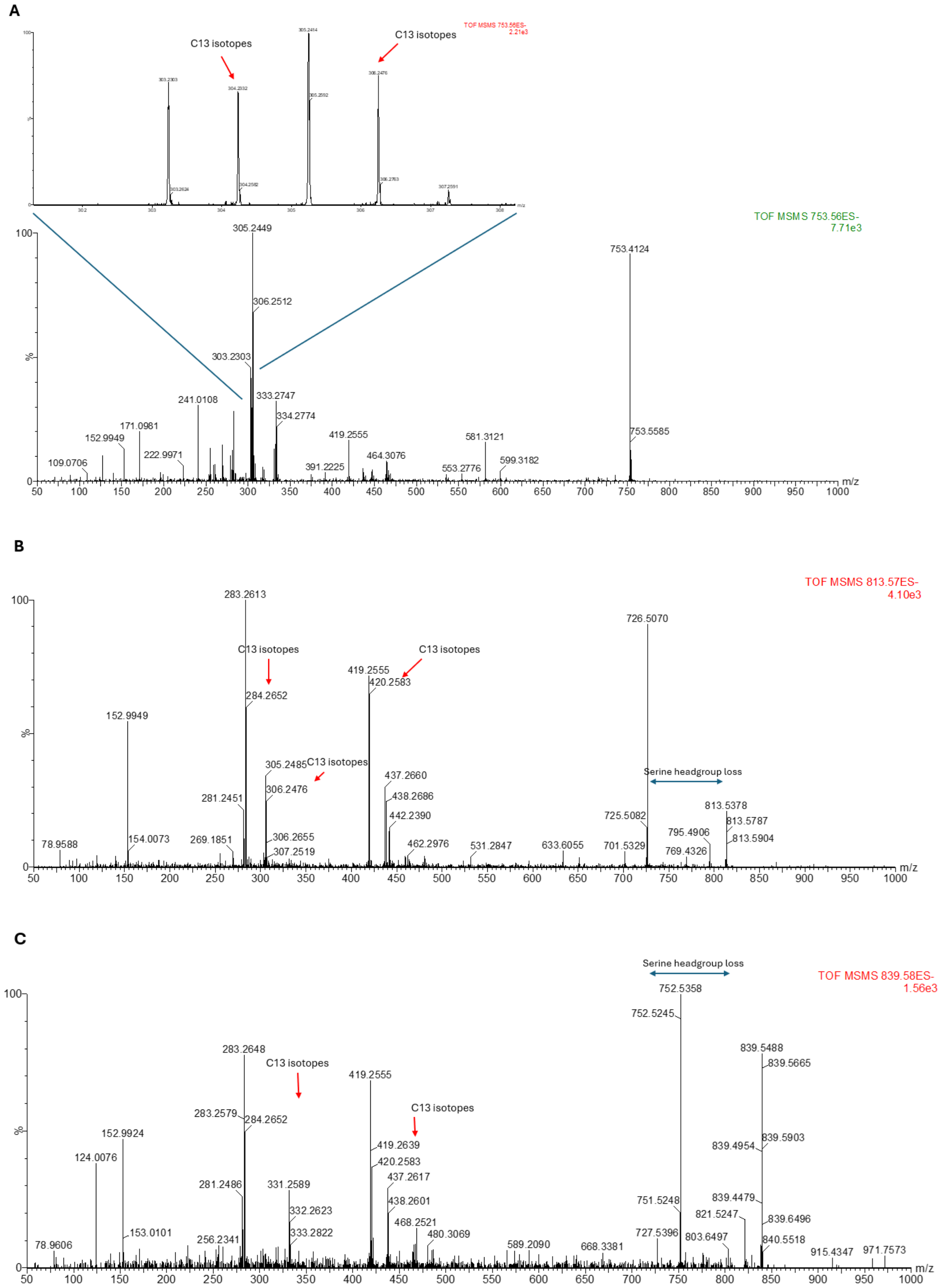

Supplementary Figure 2: MSMS spectra of ^{13}C isotopes. (A) ^{13}C isotope of PE(O-38:4) – PE(O-18:0_20:4) PE(O-18:1_20:3). **(B)** ^{13}C isotope of PS 38:3 – PS(18:0_20:3). **(C)** ^{13}C isotope of PS 40:4 – PS(18:0_22:4).

Supplementary Table 1: Subgroup analyses based on grade and stage.

Benign N=14

Grade=49: G1=15, G2=19, G3=15, unknown=1

Stage=50: stage 1a=20, stage 1b=16, stage 2=4, stage 3=8, stage 4=2

**GRADE (n)**	1st comparator<br>Correctly identified (%)	2nd comparator<br>Correctly identified (%)
Grade 2/3 (34) vs benign (14)	93.0	91.2
Grade 3 (15) vs benign (14)	93.0	87.0
Grade 1 (15) vs benign (14)	85.7	80.0
**STAGE**		
Stage 2-4 (14) vs benign (14)	93.0	93.0
Stage 1b-4 (30) vs benign (14)	92.9	90.0
Stage 1 (36) vs benign (14)	85.7	86.1
Stage 1a (20) vs benign (14)	75.0	85.7
